# Development of a competency-based clinical assessment instrument for exit level Oral Hygiene students at the University of Western Cape

**DOI:** 10.1186/s12903-022-02498-3

**Published:** 2022-10-24

**Authors:** M. Naidoo, P. Brijlal, R. Cader, N. A. Gordon, C. A. Rayner, K. Viljoen

**Affiliations:** Faculty of Dentistry, Department of Oral Hygiene, University of Western Cape, Cape Town, South Africa

**Keywords:** Clinical assessment, Instrument development, Oral hygiene

## Abstract

Seminal to the process of a health sciences curriculum evaluation is the periodic review of clinical assessment instruments that measure competency. An assessment of quality is facilitated by using a well-structured, authentic and reliable instrument. This process rests on designing and measuring the instrument against a sound framework and validating it for scientific merit. This paper documents the pedagogy and the process taken in developing an improved formative competency-based assessment instrument for the final year students of the Bachelor of Oral Health program (BOH) at the University of the Western Cape (UWC).

Methods: A qualitative research study design employing the Nominal Group Technique (NGT) was used as a method for gaining small group consensus on the clinical assessment instrument for exit level Oral Hygiene (BOH3) students within the parameters of assessment principles. The key contributors to the instrument development process were the academic staff of the Department of Oral Hygiene, involved in clinical teaching and assessment of student competency.

Results: The domains of ethics and professionalism, patient assessment, diagnosis, treatment planning and implementation was identified as the core elements in the assessment. The principles of assessment, which include, alignment with outcomes, feedback, transparency and validity, were used to guide the instrument development. The assessment criteria were cross examined for alignment to the learning outcomes of the module and the program whilst formative feedback was foregrounded as a central feature to support student learning and progress monitoring. Transparency was obtained by providing students access to the instrument before and after the assessment including the written feedback on their performance. The instrument embodied a range of criteria to be assessed rather than on the awarding of a cumulative score. This allowed for the identification of the criteria or domain within which a student is struggling or excelling. Consensus on the instrument design was achieved using the NGT phases throughout the instrument development process including the weighting of the domains and grading. This level of engagement together with the application of scientifically sound assessment principles contributed to the validation of the instrument.

Conclusion: The development of a competency-based assessment instrument was the result of a structured, collaborative and scientifically engaged process framed around specific assessment principles. The process culminated in the development of a formative competency-based clinical assessment instrument that was fit for purpose in the Bachelor of Oral Health program.

The Nominal Group Technique served to be a valuable approach for small group consensus in developing the instrument. It served to promote individual perspectives and to generate debate and group discussion between academics that were proficient in clinical teaching and, finally to facilitate group consensus on the instrument structure and system for administration.

**Supplementary Information:**

The online version contains supplementary material available at 10.1186/s12903-022-02498-3.

## Introduction

Seminal to the process of a health sciences curriculum evaluation is the review of assessment instruments that measure competence. Assessment of competence is an interpretative undertaking with inferences extending to students, instructors, and the institution. Competency has been expressed as the ability to implement or employ applicable knowledge, skills, and capabilities necessary to effectively accomplish tasks in a well-defined work-place setting [1]. An assessment of competence is a process of collating evidence to make judgements about a student’s level of learning and competence. Importantly, it informs strategies aimed at improving learning and teaching and which ultimately reflects the quality and success of the educational programme [2, 3].

A quality assessment is facilitated by using a well-structured instrument [4]. The process of developing an authentic and reliable instrument rests on designing and measuring it against a sound framework and validating it for scientific merit. An important aspect in validation is the engagement process undertaken with key role players to get consensus on the design, criteria, and outcomes to be assessed. This paper documents the pedagogy and the process undertaken in developing a formative competency-based assessment instrument for Oral Hygiene students registered in the Bachelor of Oral Health program (BOH) at the University of the Western Cape (UWC).

This research will contribute to the body of knowledge on competency-based assessments and assessment instrument development. Importantly it contributes to the quality assurance process of clinical assessment development and the broader processes of curriculum evaluation for the BOH program. Next, the BOH program will first be contextualised indicating the need for developing a competency-based assessment that is aligned to pedagogical imperatives followed by a description of the phases undertaken in developing the assessment instrument.

## Background

Curriculum review is an inherent feature of the BOH programme involving an iterative process of refining and evaluating over the years. Assessments for the different year groups are also reviewed periodically to ensure continued alignment with institutional best practices. This process of evaluation draws on international literature, the UWC graduate attributes and institutional policy documents, the Faculty of Dentistry Assessment and Moderation Protocols [5], the Assessment in Higher Education policy, (CHE, 2016) and is embedded in the core competencies guiding training in Oral Hygiene. These documents promote the employment of appropriate formative and summative assessment activities within an outcomes-based higher education environment [6]. As such assessments are developed based on principles entrenched within these documents and includes tenets such as credibility, transparency, academic integrity, and the promotion of social justice to all students [7]. Ultimately, the purpose of curriculum evaluation and assessment is to ensure that the UWC oral hygiene graduate possesses the competence required from the oral hygienist of the twenty-first century, who in South Africa, is able to practice as an independent practitioner.

The BOH program has a defined set of clinical competencies that are reflective of the knowledge, skills and values that must be achieved to deem the oral hygienist as competent for practice [8]. In order to assess the execution of these competencies an assessment instrument aligning curriculum outcomes and detailed competencies that will coalesce and regulate the entry level competencies with that of hygienists globally is needed [9]. Assessment of these competencies will enable the clinical teacher to gauge a students’ ability to meet or exceed the set criteria in the context of holistic patient care. The intimidating nature of the clinical environment along with the complexity of patient care provided a sound argument for the adoption of several models to inform teaching and assessing of competency. Previous instruments used in the BOH program were designed using a competency-based assessment approach as it offered more accountability, flexibility and learner centeredness [10]. Whilst this approach was used to guide students in their formative learning, a final decision to judge clinical competence was based on comprehensive clinical examinations. Miller’s pyramid [11] which is central to guiding assessments in the program served to map how teaching and learning could be scaffolded from classroom-based learning to preclinical and ultimately independent and holistic patient care in the various clinical platforms where students treat patients. The one-minute preceptor model, a student-centred approach, which is used to shape the clinical conversation and feedback between the student and clinical teacher [12] has also been applied as a teaching and learning model in the oral hygiene clinical environment.

In its most recent evaluation (2018–2021), the department started the process of reviewing the exit level clinical practice modules of the BOH programme as well as the assessment instruments. The focus in the current study was on the assessment tool for the exit level module, Clinical Practice 3 (CLP300). This is a 40-credit module, that is, 400 notional learning hours, that comprises of theory, a small proportion of pre-clinical procedures and a significant allocation of time for clinical care of patients. In this module, the theory and the clinical teaching and training of the three years coalesce. The student should demonstrate competence in clinical practice in preparation for the world of work.

The general notion of using a clinical examination mark as an indication of competence was seen as problematic as this was an inherent contradiction within the current approach to competency-based assessment in the clinical environment. The diversity of patients, reliability of teacher assessment scores, student attributes, emotions and previous experiences of summative assessments [13] were amongst the arguments for this change. In addition, this notion of reviewing current assessment practices is in line with a need for clinical teachers to be innovative in providing evidenced based and reliable assessment instruments within a rapidly evolving academic and health context.

Assessment instrument development requires academic integrity and to achieve this the mapping of an assessment instrument must be informed by evidence that includes frameworks to validate the process [14]. In the next section the core elements in assessment instrument development will be discussed and includes the value of a competency-based approach, assessment alignment with curriculum outcomes, feedback, and transparency and validation of assessment instruments.

### A competency-based assessment approach

Assessment has been defined as “*the systematic collection and analysis of information to improve student learning*”, that allows for the recognition of effective teaching and learning practices to reflect one’s pedagogy and to record evidence of meaningful learning [15]. Dental education had witnessed a paradigm shift from a predominantly content-based curriculum to one that is competency focused [16]. The primary undertaking of competency assessments is to produce an entry-level professional capable of independent function. Competency-based assessment implements have extensive acceptance as a measure for assessing a student’s task- related ability [17] and offers more accountability, flexibility, and importantly, it is learner-centred [18]. It encompasses gathering of evidence to validate judgements regarding the student’s progress, for the development of proficient standards and the mastery of skills. It also reflects the student’s ability to translate theory into clinical practice and demonstrate this ability in a work-based context such as the clinic [19]. However, the potential to assess all the dimensions that undergraduates are anticipated to demonstrate is a major challenge. To ensure transparency for both students and teachers, it is imperative that the assessment is aligned to learning objectives and module outcomes.

### The value of aligning assessments with learning objectives

Aligning assessments with learning objectives and outcomes will not only provide insights on the extent to which the outcomes have been achieved but it will also facilitate student learning whilst ensuring validity and accountability [20]. Outcomes and objectives refer to what is expected of the student to know and be able to do whilst the assessment will reveal to what extent the student has achieved those goals [21]. If assessment criteria are mismatched with the outcomes, measuring the achievement of learning outcomes as well as student progress can become problematic. Hence explicit assessment criteria are seminal to the assessment instrument to create transparent and explicit achievement benchmarks for individual learning outcomes. It serves to postulate how tasks can be assessed [22].

The assessment criteria must also reflect what a student should know and be able to demonstrate as reflected in Miller’s Pyramid which differentiates between knowledge at the lower levels and action in the higher levels. A recent review of this pyramid reflects domains of Bloom’s Taxonomy [11]. The core purpose of Bloom’s taxonomy is to present the learning exemplar progression [23] and to illustrate the progression of learning into higher levels of thinking. This taxonomy is pursued during theoretical and practical assessment components in dental education such as in formative assessments.

### Formative learning and the value of feedback

Another important aspect on facilitating or supporting learning through assessment is the formative element that allows for the provision of the continuous evaluation of a student’s learning and progress [24]. This kind of assessment practice necessitates the evaluation of learning outcomes on numerous occasions and facilitating content and skill-specific evaluation [25]. An important component of formative assessment is feedback, which has been defined by Boud & Molloy  as “*A process whereby learners obtain information about their work in order to appreciate the similarities and differences between the appropriate standards for any given work, and the qualities of the work itself, in order to generate improved work*”. These authors propose that a new paradigm shift in feedback should incorporate a more constructivist and student-oriented approach [26].

When developing an assessment instrument, ideally it should provide the assessor with sufficient information to gauge a student’s progress, and simultaneously offer the student enough feedback to leverage their learning. Various models of feedback have been advocated, with each possessing their individual merits and challenges. Irrespective of the model implemented, feedback should follow specific guidelines [27]. The repeated emphasis and focus in literature on student learning with regards to addressing learning gaps, the role of this practice in teaching and learning as well as the role of feedback for improvement has been noted. However, research has also alluded to the noticeably reduced focus on the confluence of each of these aspects [28].

### Validating assessment instruments

In assessment development, validity is generally measured by how well the instrument measures what it intends to measure and whether the decision about a candidate’s competence is justified [29]. The authors argue that it is necessary to determine the conditions that undermines the integrity of the assessment, by exploring explanations that account for the good or bad performance and finally channelling this information into the review process so as to minimize errors when assessing. They further highlight several ways to ensure reliability and validity. Firstly, in developing an instrument, there must be evidence of how validity and reliability has been tested and built into the design and use of the instrument. Secondly instruments must be reflected on and reviewed periodically to increase validity. Thirdly that the instrument must be designed for the group it was intended and not expect similar outcomes for a different group. Fourthly that assessments should be mapped against policy documents, such as, outcomes of a curriculum. Fifthly that the instrument be subjected to the input from experts (face and content validity). Lastly, one should not mistake authenticity for validity as even a well-developed model is no guarantee of the quality of the items. Constructs need to be defined accurately so that it truly measures the skills and knowledge it is intended to measure [29].

The reviewing of the existing assessment instrument was thus intended to consolidate and build on the strengths of the current system but work towards developing an integrated and authentic model of clinical teaching and assessment that would meet the criteria of being outcomes-based and student-centred with dimensions of being patient-centred. This paper documents the pedagogy and the process involved in developing a formative competency-based assessment instrument making it more robust and fit for purpose for exit level (BOH3) students. The authors proposed that a purposely representative model of knowledge and skills employing a judiciously constructed “blueprint” based on a structured process be developed and implemented [14].

## Methodology

### Study design, context and participants

A qualitative research study design employing the Nominal Group Technique (NGT), which is a consensus gaining approach, was used by a designated group of academics to develop an assessment instrument aligned to the principles of assessment for exit level Oral Hygiene (BOH3) students. The study context was the Bachelor of Oral Health program located within the Department of Oral Hygiene at the University of Western Cape. The purposefully designated group was the six permanent academic staff (*n* = 6) within the Department of Oral Hygiene, tasked for the reviewing and designing of the assessment instrument. These staff members with expertise in clinical teaching and assessment, were also involved in previous instrument development for the same module. The lead researcher facilitated the scheduling, facilitation, the recording and documenting of the group consensus discussions. The review of the assessment instrument is part of a registered Department project for curriculum review (BM/16/5/9) which focuses on evaluating teaching and learning in the BOH program.

The NGT facilitated the gathering of qualitative data [30] and was the technique of choice as it *“seeks to provide an orderly procedure for obtaining qualitative information from target groups who are most closely associated with a problem area*” [31]. The study was aligned to the NGT [32] which foregrounds inclusivity and equal contribution when gaining consensus and typically involves several steps: the individual generation of ideas, recording of all ideas and suggestions, group discussion of the ideas, preliminary selection of pertinent ideas and reconvening with group discussion until consensus is reached and a final decision made. In this study, the NGT process started with an initial meeting on an online platform with the academics which allowed for group members to initially present their own perspectives on the existing assessment instrument, rather than as a group. Data from this meeting framed the agenda for the next meeting and were considered within the context as starting points for discussion. Multiple online and face to face group discussions that were iterative in nature allowed members to go back and forth with the development of the instrument. The group members were allowed equal opportunity to express their views and debate on the suggestions of all members. Where there were differences of opinion, individual members were allowed further opportunity to engage in discussion so as to reach consensus from all members. Subsequently, group consensus was reached and the instrument was refined as members became better informed through discussions and consultation. All meetings were recorded and minuted. Audio recordings and typed notes served as data and was analysed by formulating themes which were categorized according to ‘layout and flow of the instrument’, ‘criteria and wording for each domain of assessment’, ‘grading and weighting’ and ‘general information’.

The existing assessment instrument, the literature and group consensus was used to guide the identification of the final domains for the development of the new instrument.

The development of the assessment instrument was conducted in five phases and was informed by the assessment principles of the University and Faculty [5], which include, alignment of assessment criteria with learning outcomes, feedback and transparency and validity.

These phases highlighted below will be discussed in detail as part of the reporting and discussion:

- Phase 1: establishing the key role players and delineating the purpose of the assessment.
- Phase 2: reviewing of the previous assessment instrument against the assessment principles, learning outcomes and literature.
- Phase 3: refining the instrument and gaining consensus on the criteria.
- Phase 4: gaining consensus on the grading and weighting of each domain.
- Phase 5: alignment of the assessment instrument to the assessment principles.

## Results and discussion

Phase 1 focused on establishing the key role players and delineating the purpose of the assessment. The NGT was instituted with 6 academic staff who were involved with clinical teaching and assessment in the BOH program and their role and responsibilities in the review process was established. In order to facilitate an efficient assessment review process, it was beneficial that all role-players understood their individual roles and responsibilities. According to the South African Qualifications Authority (SAQA) [33] document, these roles highlight the importance of evidence and quality assurance as reflected in the following clause: “*All relevant role-players must be able to provide evidence of the development and moderation of assessment tasks and processes, and that these tasks and processes are aligned with National Policy for Designing and Implementing Assessment as well as sectoral policies derived from the National Policy”*.

The active commitment of all staff during the NGT process resulted in the attainment of information that was not dominated by any single group member nor the facilitator. An important benefit of the NGT was that it allowed for collegial collaboration and consensus through various discussions particularly in view of the many different lecturers teaching on the clinical practice module. The NGT allowed for the building of trust, the instilling of ownership, promotion of commitment to the project as well as a means towards achieving calibration. The existing instrument was then discussed in terms of its fit for purpose and the need to review the instrument against assessment principles and learning outcomes was established.

The purpose of the assessment was established as follows:

- To determine the oral hygiene student’s ability to assess their patient, make a diagnosis, develop a treatment plan, implement and evaluate treatment whilst observing core principles of ethics and professional behaviour,
- To evaluate the clinical competence of a student according to the oral hygiene scope of practice and their readiness for independent clinical practice,
- To inform further training needs towards the achievement of clinical competency,
- To grade student clinical performance in competence terms.
- To assess the student’s ability to demonstrate clinical reasoning and critical thinking skills during patient management.,
- To establish the overall achievement in the translation of knowledge to practice.

Phase 2 involved the reviewing of the previous assessment instrument against the learning outcomes of the program, the assessment principles and literature in order to identify areas for revision and improvement. Local and national Oral / Dental Hygiene study programs were explored to identify assessment approaches and competency domains that are common or different from the Oral Hygiene program (BOH) at UWC. An electronic search of the databases was conducted using keywords such as, competencies, oral hygiene, assessment, clinical education, and competency domains. A consensus was reached amongst the group on the domains to be included in the development of the assessment instrument.

### Identification of assessment domains

Five main domains were identified to assess clinical competence based on the provision of holistic care and framed within the scope of the Oral Hygienist. These domains included ethics and professionalism; patient assessment; diagnosis and treatment planning and treatment implementation. The domains were not different from the previous assessment instrument used. However, its application was to vary in terms of refined criteria and weighting in the revised instrument.

The first domain of *ethics and professionalism* is consistently identified as an important competence. It is featured here as an individual domain but it is also assessed as an implicit feature that is underpinned in all the domains. With the increasing emphasis on teaching and assessment of professionalism within the Oral Hygiene curriculum, this aspect is assessed as a core competency that is reflected as a mark denoting the overall level of ethics and professionalism displayed by the student. The second domain, on *patient assessment* relates systematic collection, documentation and analysis of the information attained through a range of interventions in order to make a diagnosis and draft a corresponding patient management and treatment plan. The process includes a patient interview to gather social, medical and dental histories, dietary analysis, general health appraisal, comprehensive clinical extra and intra-oral, the charting of dental and periodontal status, risk assessments for caries and periodontal disease and radiographic evaluation. The third domain is that of *diagnosis*. It focuses on the ability of the student to perform an evidence-based analysis and interpretation of the assessment performed in order to make a summation of the patient’s oral health status as well as the recognition of the etiological factors contributing to ill health. The fourth domain is that of treatment planning which involves the selection of interventions in response to the diagnosis and specific health / oral health problems the patient presents with. This domain includes the plan of action within a comprehensive care plan that is cognisant of the etiological factors that must be addressed. It also includes referrals for treatment procedures not within the scope of the Oral Hygienist. The fifth domain focuses on the implementation of educational, preventive, and therapeutic oral care services to support and attain optimal oral health for the patient. It includes the process of performing an evaluation of the patient in response to intervention. Throughout the patient management process, the student must demonstrate the ability to translate knowledge into practice, and use critical thinking skills and clinical reasoning.

### Alignment of outcomes to assessment criteria

Phase 3, involved the refining of the instrument and gaining consensus on the criteria that was created to assess in each domain. Thereafter the criteria within each domain were checked for alignment with the learning outcomes. The outcomes include the knowledge, skills, and values to be attained and as stipulated in the module descriptor of the clinic practice module. These learning outcomes were developed on the basis of the Oral Hygiene Scope of practice (HPCSA) [34] and SAQA exit level outcomes [33]. The process of alignment (Table 1) was guided by the principles described by (Gosling and Moon, 2002) [35].

**Table 1:** Alignment of Guiding Principles to the current Assessment

*Guiding Principle (Gosling and Moon, 2002)*	*Alignment to Current Assessment Strategy*
*“All learning can be expressed as demonstrable outcomes to be achieved”*	1. Ethics and Professionalism: Demonstrated as an observed behaviour 2. Theory translated into practice and directly observed as a skill 3. Critical think and clinical reasoning observed during clinical decision making in assessment and diagnosis
*“All domains are described in terms of their learning outcomes and assessment criteria”*	1. The assessment domains and sub-domains have detailed outcomes and assessment criteria as displayed in (Appendix 1)
*“The type and number of learning outcomes and assessment criteria form the basis for assigning a number of credits and a level to a particular domain”*	1. Based on the learning outcomes and criteria, appropriate weighting were attached to specific outcomes
*“Learning outcomes need to be clear and unambiguous”*	1. Clear explicit outcomes and criteria are detailed and stipulated in the assessment tool (Appendix 1)
*“Learning outcomes set out the necessary learning, which represents the minimum requirement for a pass grade on the unit”*	1. The specified standard as required according to the criteria to demonstrate clinical competence is clearly stated in the assessment after collegial agreement
*“Assessment criteria should specify how a satisfactory performance of the learning outcomes would be demonstrated”*	1. The identified criteria of a range between 5 to 7 depending on assistance required for achievement allows for the satisfactory achievement of a specified outcome
*“Learning outcomes should contribute to the transparency of the overall qualification gained by enabling students, parents, prospective employers and other educational professionals to understand exactly what has been learned in order to achieve a passing grade”*	1. The learning outcomes are made available and discussed with all stakeholders to understanding and transparency

The assessment criteria were further refined following the interrogation of the assessment instrument which contributed towards its validation. The process allowed for opportunity to gain consensus on areas revealed to be vague, ambiguous and biased.

The level descriptors in the assessment criteria included: “not achieved”, “partially achieved”, “achieved” and “exceeds expectations” with corresponding scores ranging from 1–10.

Appendix 1 highlights the criteria and corresponding scores that were developed to facilitate the students learning towards achieving the set outcomes.

### The weighting and grading of the assessment instrument

Phase 4 involved the gaining of consensus on the grading and weighting of each domain. The process was informed by the need to provide an assessment that was more student-centred and holistic and that embroidered on knowledge, skills and values but also allowing for the potential to judge the student’s competency in a varied range of tasks [36]. To this end, a score was to be assigned based on an inclusive decision of the student’s work. The blueprint of this clinical assessment was constructed as a rubric that was based on the five domains. The domains for the clinical assessment instrument were weighted according to the complexity of learning and the skills needed to demonstrate the level of competency. The group collectively agreed upon the weighting and scoring (Appendix 1). The assessment instrument was divided into two sections. The first section included the domains of ethics and professionalism weighted at (15%). Assessment (30%), Diagnosis (25%) and Treatment planning (25%) which totalled to 100% for part one. In part two (treatment procedures) all clinical procedures implemented was equally weighted and an average calculated. A summative total was obtained by summing up 50% of part one and part two, respectively (Appendix 1).

### Alignment to assessment principles

Phase 5, focused on aligning the assessment instrument in terms to the assessment principles. The review of the clinical assessment process as depicted in Table 2 was aligned to and located within a framework of assessment principles as set out by the Council of Higher Education [6], UWC Assessment guidelines and the Assessment and Moderation Policy of the Faculty of Dentistry [5].

**Table 2:** Alignment of Assessment Principles to Current Assessment

*Assessment Principle*	*Details*	*Alignment to Current Assessment Strategy*
Reliability	The accuracy with which an assessment measures the skill that which it is designed to measure (Butt 2010: 46)	Clear and consistent procedures were delineating to ensure that the clinical teachers implemented consistent criteria as outlined in the instrument to arrive at similar conclusions
Face Validity	The assessment task assessing its intended task	The clinical task was assessed according to the detailed outcomes and criteria
Content Validity	The assessment task correspondingly representing the envisioned scope/ learning	Outcomes and criteria set out in this assessment was aligned to learning outcomes of the corresponding clinical module (CLP 300)
Criterion Validity	The extent to which the assessment task was based upon a similar assessment Task	The continuous assessment (Formative) allowed for similar tasks and to inform future task in the clinic
Predictive Validity	The extent to which a student’s past performances in an assessment task is considered predictive or the student’s future performance in a specific area of learning	The continuous assessment (Formative) allowed for the tracking of performances in similar tasks and learning areas
Concurrent Validity	The association between the results of one assessment task when compared to that of another assessment task that focused on the same outcome in a similar learning area	The continuous assessment (Formative) allowed for the comparison of competencies in similar and similar learning areas
Construct Validity	The extent that an assessment task measures criteria that is challenging to observe directly	The domain of ethics and professionalism is often challenging to observe directly. This aspect is measured or inferred indirectly. (Example: Signed consent)
Fairness	Unbiased assessments offering students equal opportunities to demonstrate their competence	All students were assigned with unknown patients, assessed in the same clinical context employing the same set out outcomes and criteria
Cognitive Complexity	Representation of a variety of cognitive levels	The domains represented in this assessment was representative of various cognitive levels according to Bloom’s Taxonomy
Generalisability and Transferability	The extent to which student abilities can be transferred and the application of knowledge and skills to different contexts	This was a work-place based assessment that can be transferred to similar contexts outside of the institute
Authenticity of Evidence	The evidence gathered is the student’s own work	Direct clinical observation ensure authenticity of the evidence gathered
Meaningfulness	A valuable learning experience attained in the assessment process	The assessment task was clearly linked to the learning outcomes of this module, thereby integrating teaching and learning
Balance	The time allocated to complete the assessment task was in balance with the complexity of the task	The assessment task –time was allocated according to its complexity and the students were provided with a dental assistant
Transparency	The extent to which the assessment plans are shared with the recipient	The expected learning outcomes, the assessment instrument, the assessment criteria as well as the mark allocation was shared with the students

### Validation of the assessment instrument and the development review process

The main strength of the review and development process of the assessment instrument is that a structured and collaborative process was followed within a context of multilevel consultation. Consultation was with literature, expert academics (clinical teachers) who are involved in assessment as well as the students. Consensus at interim points assured some degree of calibration as the staff using the instrument are the same as the group involved in the development of the instrument. To some extent this process assured reliability in that the instrument would be least affected by influences from different clinical teachers.

Transparency was a further assessment principle that was incorporated in the assessment development process and it relates to the extent to which the assessment plans are shared with the recipient, in this case, the students being assessed. In this project, it began with the sharing of the expected learning outcomes, the assessment instrument, the assessment criteria as well as the mark allocation. The act of transparency also contributed to the validation process of the assessment.

## Conclusion

The clinical assessment instrument for the exit level BOH3 students was developed and validated through a rigorous process using the Nominal Group Technique. This technique was suitable in gaining individual perspectives, to generate debate and group discussion between academics that were proficient in clinical teaching and, finally to facilitate group consensus on the structure and system for administration. The instrument reflected five domains namely, ethics and professionalism, patient assessment, diagnosis, treatment planning and treatment implementation were seminal areas being assessed. The criteria provisioned to facilitate the instrument development were aligned to the assessment criteria, the outcomes of the clinical practice module, the exit level outcomes of the program, as well as the scope of profession of the Oral Hygienist. The assessment instrument which was deemed fit for purpose in assessing clinical competency in the Oral Hygiene program was developed being mindful and encompassing of the assessment principles embedded in policies of the University, Faculty and as set out by the Council on Higher Education.

## Supplementary Information

**Additional file 1.** 

## Data Availability

The dataset used and/or analysed during the current study is available from the corresponding author on reasonable request. All data is stored at The University of western Cape, South Africa. All primary data collected in this study was in the form of written documentation, this primary data will be stored in the Faculty of Dentistry, Department of Oral Hygiene. The objective of this is to guarantee security and integrity of this primary data set. The overall responsibility for this primary data will be according to the policies of the University of Western Cape, within the Head of Department of Oral Hygiene.
