# Supplementary material for: Development of a competency-based clinical assessment instrument for exit level Oral Hygiene students at the University of Western Cape
Source: BMC Oral Health. 2022 Oct 24;22:452. doi: 10.1186/s12903-022-02498-3 (PMC9590136; doi:10.1186/s12903-022-02498-3)
Supplement: Supplementary file 1 — Additional file 1. [file 12903_2022_2498_MOESM1_ESM.docx]

APPENDIX 1: COMPETENCY-BASED CLINICAL ASSESSMENT INSTRUMENT

| **Learning outcome and Associated Assessment Criteria** | **Not Achieved Required substantial**  **guidance to contextualize ethical principles)** | **Partially Achieved Required moderate guidance**  **to contextualize ethical principles)** | **Achieved**  (**Required minimal guidance to contextualize ethical principles)** | **Exceeds Expectation (Required no guidance to contextualize ethical principles)** |
| --- | --- | --- | --- | --- |
| ***Ethics and Professionalism***  ***Learning outcome***: The student should be able to: Demonstrate personal characteristics within the parameters of ethical regulatory standards and professionalism during the provision of oral health care services in clinical practice sessions.  **Assessment criteria:**  **The student can:**   - **Demonstrate** an image of professionalism and maturity during clinical rotations by **arriving timeously**, preparing **workstation, greeting** colleagues, supervisors and patients. - **Provide** unbiased **information** to allow the patient to make decisions. - **Obtain Informed consent**, ensuring that the patient, parent, caregiver or guardian gives authorization for a procedure or comprehensive treatment plan. - **Confidentiality,** to ensure privacy in the manner in which information is gathered, stored and shared with other health care professionals. - **Prevent** and **remove….** of harm by using universal precautions during all aspects of treatment and when educating the patient in homecare. - **Provide** competent and timely oral care considering the **needs, desires, and values** of the patient. - **Practice** justice by providing patient care without **discrimination** against race, creed, sex or nationality or socio-economic status. | The student **did not display** awareness of the ethical regulatory standards and professionalism during the provision of oral health care services. | The student displayed **limited awareness** of the ethical regulatory standards and professionalism during the provision of oral health care services.  The student still needs to demonstrate consideration of the ethical perspective in an appropriate manner | The student displayed **a good awareness** of the ethical regulatory standards and professionalism during the provision of oral health care services.  The demonstration of and consideration of the ethical perspective was concise and appropriate. | The student displayed **an in- depth awareness** of the ethical regulatory standards and professionalism during the provision of oral health care services.  The demonstration of and consideration of the ethical perspective was concise and appropriate. |
| ***15%*** | 1-2 | 3-4 | 5-7 | 8-10 |

| **Assessment** | | | | |
| --- | --- | --- | --- | --- |
| **Learning outcome and Associated Assessment Criteria** | **Not Achieved (Required substantial**  **guidance to methodically investigate patient-related findings relating to the assessment component).** | **Partially Achieved (Required moderate guidance**  **to efficiently recognize and methodically investigate**  **patient-related findings relating to the assessment component.** | **Achieved** (**Required minimal guidance to efficiently recognize and**  **methodically investigate patient-related findings relating to the assessment component).** | **Exceeds Expectation (Required no guidance to producing an assessment that was appropriate and relevant displaying an evident contextualization of the assessment**  **approach.)** |
| ***Assessment***  **Learning outcome:**  **The student should be able to demonstrate** critical reasoning skills to methodically assess all patients by integrating knowledge of general, behavioural, social, life style practices, and extra-oral, intra-oral and radiographic findings, additional relevant investigations to support the assessment component of the dental hygiene process of care.  **Criteria:**   - Inquiring, collecting and documenting information on the **main complaint.** - Retrieve information in the previous **dental history** of the patient. - Document the medical history and identify conditions requiring **special considerations.** - Gather information on the patient’s life style practices and accordingly conduct **further investigations** implementing a caries risk assessment, dietary analysis and smoking cessation. - Conducting and recording findings of a thorough and comprehensive **extra-oral** examination incorporating JACCOL. - Conducting and recording findings of a thorough and comprehensive **intra-oral** examination as detailed in the dental hygiene process of care, incorporating, (BPE, PI%, bleeding%, hard and soft tissue examination and **radiographic examination**).   **Synthesis of information and methodical** presentation of all assessment findings. | The student **displayed no** evidence of: critical reasoning skills to methodically assess the patient, employing the relevant exploratory tools and approaches to complete the assessment component of the dental hygiene process of care. | The student **displayed limited** evidence of: critical reasoning skills to methodically assess the patient, employing the relevant exploratory tools and approaches to complete the assessment component of the dental hygiene process of care. | The student **displayed good** evidence of: critical reasoning skills to methodically assess the patient, employing the relevant exploratory tools and approaches to complete the assessment component of the dental hygiene process of care. | The student **displayed excellent** evidence of: critical reasoning skills to methodically assess the patient, employing the relevant exploratory tools and approaches to complete the assessment component of the dental hygiene process of care. |
| ***30%*** | 1 - 2 | 3 - 4 | 5 - 7 | 8 – 20 |

| **Diagnosis** | | | | |
| --- | --- | --- | --- | --- |
| **Learning outcome and Associated Assessment Criteria** | **Not Achieved (Required substantial**  **guidance to interpret and convey the information in a systematic manner displaying clinical decision- making).** | **Partially Achieved (Required moderate guidance to interpret and convey the information in a systematic manner displaying clinical decision- making).** | **Achieved**  (**Required minimal guidance to interpret and convey the information in a systematic manner displaying clinical decision-making).** | **Exceeds Expectation (Produced an in-depth reflection of all assessment findings to critically analyses, and made a deductive definitive a differential**  **diagnosis.)** |
| ***Diagnosis:***  **The learning outcome:**  **The student should be able** to demonstrate critical thinking and clinical reasoning skills to arrive at a deduction:  **Assessment criteria:**  **The student can…..**  To provide:   1. A **definitive periodontium diagnosis according to the new AAP Classification.** 2. **Hard tissue diagnosis of caries.** 3. Identify associated etiological factors. 4. The student when applicable must also be able to demonstrate clinical reasoning to make a differential diagnosis, which is the practice of considering the possibility of one condition to that of other condition that will probably account for the present findings.  - **Analyse** and contextualize the assessment findings to   **formulate** a definitive and/or differential diagnosis.   - Provide a j**ustification** for the diagnosis by referring to recorded findings and evidence based practice. - Identify predisposing **etiological** risk factors. - **Recognize** the need for further investigations including a comprehensive periodontal screening, caries risk assessment, dietary analysis, smoking cessation, and further diagnostic radiographs. - **Collaborate** with other health care providers to provide interdisciplinary oral health care. | The student displayed a **no ability** to analyse the assessment findings and assimilate this information to provide a definitive and differential diagnoses. | The student displayed a **limited ability** to analyse the assessment findings and assimilate this information to provide a definitive and differential diagnoses. | The student displayed a **no ability** to analyse the assessment findings and assimilate this information to provide a definitive and differential diagnoses. | The student displayed a **no ability** to analyse the assessment findings and assimilate this information to provide a definitive and differential diagnoses. |
| ***25%*** | 1 - 2 | 3 - 4 | 5 - 7 | 8 – 10 |

| **Treatment Planning** | | | | |
| --- | --- | --- | --- | --- |
| **Learning outcome and Associated Assessment Criteria** | **Not Achieved (Required substantial guidance to**  **contextualize and justify preventive, promotive, therapeutic and maintenance interventions included in the comprehensive treatment plan).** | **Partially Achieved (Required moderate guidance to**  **contextualize and justify preventive, promotive, therapeutic and maintenance interventions included in the comprehensive treatment plan).** | **Achieved**  (**Required minimal guidance to contextualize and justify preventive, promotive, therapeutic and maintenance interventions included in the comprehensive treatment plan).** | **Exceeds Expectation (Produced a comprehensive treatment plan was substantiated by evidence based practices and understandings which exceeded the course**  **expectations)** |
| ***Treatment Planning***  The student should demonstrate the ability to formulate a comprehensive, sequential treatment plan according to organized visits by applying the complete assessment findings, provides a rationale for the treatment plan, and describes the treatment plan in the context of the specific patient.  **Assessment criteria:**  **The student can**   - **Formulate** a comprehensive plan of treatment and a written treatment plan based on the diagnosis and social context. - Include **preventive, therapeutic, and educational**, oral care services tailored to the patient’s needs and expectations. - Formulate the treatment in line with the **best available evidence** - Permit the **active involvement** of the patient/and/or parent, caregiver and guardian. - Developed a treatment plan that is **sequential** and prioritized according to **organized visits** and the patient needs.   Include **referrals** in the treatment plan where applicable. | The student displayed **no** evidence to display the ability of formulating a comprehensive treatment plan in sequential organized visits. | The student displayed **limited** evidence to display the ability of formulating a comprehensive treatment plan in sequential organized visits. | The student displayed **good** evidence to display the ability of formulating a comprehensive treatment plan in sequential organized visits. | The student displayed excellent evidence to display the ability of formulating a comprehensive treatment plan in sequential organized visits. |
| ***30%*** | 1 - 2 | 3 - 4 | 5 - 7 | 8 - 10 |

Part One (50%)

| ***Part Two - Clinical Procedures (50%)*** | | | | |
| --- | --- | --- | --- | --- |
| **Learning outcome and Associated Assessment Criteria** | ***1-2***  ***Not Achieved*** | ***3-4***  ***Partially achieved*** | ***5-7***  ***Achieved*** | ***8-10***  ***Exceeds expectations*** |
| The student must demonstrate the ability to deliver dedicated treatment that is aligned to patient needs and expectations, diagnosis and goals. The treatment should include preventive, therapeutic and educational procedures according to the dental hygiene process of care to maintain an optimum state of oral health.  **Assessment criteria:**  **The student can**   - **Utilize** mandated infection control procedures. - Use **accepted clinical techniques** within the HPCSA scope and practice of oral hygienist. - Incorporate diagnostically acceptable tools such as **radiographs, CAT, Dietary Analysis, smoking cessation.** - Implement **instrumentation,** avoiding trauma to the patient. - Performed treatment according to the **treatment pla**n and **modify** as required. - Apply and select the **appropriate materials** for the completion of specified procedures. - Constant **monitoring** of change in the context of assessment and treatment tools and adjust as required. - **Evaluate** the efficacy and practicality of the treatment plans and adjusted accordingly.   **Timeous** implementation and completion of procedures. | The student displayed no evidence to implement holistic treatment by means of mandated clinical techniques and infection control measures. Excessive tine spent on simple procedures.  However the following will be taken into account i.e. the type of patient and the complexity of the case | The student displayed limited evidence to: implement holistic treatment by means of mandated clinical techniques and infection control measures. Spends reasonable time on procedure with assistance e.g. prompting the student to finish or assisting the student with the procedure | The student displayed with minimal guidance acceptable evidence to: implement holistic treatment by means of mandated clinical techniques and infection control measures. Spends reasonable time on procedure with guidance from the supervisor | The student displayed excellent evidence to: implement holistic treatment by means of mandated clinical techniques and infection control measures. |
| **50%** | **1 - 2** | **3 - 4** | **5 - 7** | **8 - 10** |
